# Animal trypanosomosis eliminated in a major livestock production region in Senegal following the eradication of a tsetse population[Fn FN1]

**DOI:** 10.1051/parasite/2024010

**Published:** 2024-03-06

**Authors:** Momar Talla Seck, Assane Guèye Fall, Mamadou Ciss, Mame Thierno Bakhoum, Baba Sall, Adji Marème Gaye, Geoffrey Gimonneau, Mireille Djimangali Bassène, Renaud Lancelot, Marc J.B. Vreysen, Jérémy Bouyer

**Affiliations:** 1 Institut Sénégalais de Recherches Agricoles, Laboratoire National de l’Elevage et de Recherches Vétérinaires route du Front de Terre 11500 Dakar – Hann Sénégal; 2 Ministère de l’Elevage et des Productions Animales, Direction des Services Vétérinaires Sphère Ministérielle Ousmane Tanor Dieng 20000 Dakar – Diamniadio Sénégal; 3 CIRAD, UMR INTERTRYP 37, avenue Jean XXIII BP 6189 12900 Dakar-Etoile Sénégal; 4 INTERTRYP, Univ Montpellier, CIRAD, IRD Campus International de Baillarguet 34398 Montpellier France; 5 ASTRE, CIRAD 34398 Montpellier France; 6 ASTRE, Cirad, INRAE, Univ. Montpellier, Plateforme Technologique CYROI 97491 Sainte-Clotilde, La Réunion France; 7 Insect Pest Control Laboratory, Joint FAO/IAEA Programme of Nuclear Techniques in Food and Agriculture Wagramerstrasse 5 1400 Vienna Austria

**Keywords:** Trypanosome, Seroprevalence, Case-control study, Eradication, Sterile insect technique

## Abstract

African animal trypanosomosis (AAT) was one of the main disease-related constraints to the development of intensive livestock production systems in the Niayes region of Senegal, a 30 km wide strip of land along the coast between Dakar and Saint-Louis. To overcome this constraint, the Government of Senegal initiated an area-wide integrated pest management programme combining chemical control tactics with the sterile insect technique to eradicate a population of the tsetse fly *Glossina palpalis gambiensis* Vanderplank, 1949 (Diptera, Glossinidae) in this area. The project was implemented following a phased conditional approach, and the target area was divided into three blocks treated sequentially. This study aims to assess the temporal dynamics of the prevalence of *Trypanosoma* spp. during the implementation of this programme. Between 2009 and 2022, 4,359 blood samples were collected from cattle and screened for trypanosomes using both the buffy coat and ELISA techniques, and PCR tests since 2020. The seroprevalence decreased from 18.9% (95%CI: 11.2–26.5) in 2009 to 0% in 2017–2022 in block 1, and from 92.9% (95%CI: 88.2–97) in 2010 to 0% in 2021 in block 2. The parasitological and serological data confirm the entomological monitoring results, *i.e.*, that there is a high probability that the population of *G. p. gambiensis* has been eradicated from the Niayes and that the transmission of AAT has been interrupted in the treated area. These results indicate the effectiveness of the adopted approach and show that AAT can be sustainably removed through the creation of a zone free of *G. p. gambiensis*.

## Abstract (fr)

La trypanosomose animale africaine (TAA) était l’une des principales contraintes pathologiques au développement de systèmes de production animale intensifs dans les Niayes du Sénégal, une bande de terre large de 30 km longeant la côte entre Dakar et Saint-Louis. Pour surmonter cette contrainte, le Gouvernement du Sénégal a lancé un programme de lutte intégrée à l’échelle de la zone combinant lutte chimique et technique de l’insecte stérile pour éradiquer une population de *Glossina palpalis gambiensis* Vanderplank, 1949 (Diptera, Glossinidae). Le projet a été mis en œuvre selon une approche conditionnelle progressive, et la zone cible a été divisée en trois blocs, traités de manière séquentielle. L’objectif de cette étude était d’évaluer la dynamique temporelle de la prévalence de *Trypanosoma* spp. au cours de la mise en œuvre du programme. Entre 2009 et 2022, 4 359 échantillons de sang ont été prélevés sur des bovins et ont fait l’objet d’un dépistage des trypanosomes à l’aide des techniques du buffy-coat et ELISA, ainsi que de test PCR depuis 2020. Dans le bloc 1, la séroprévalence est passée de 18,9 % (IC 95 % : 11,2–26,5) en 2009 à 0 % entre 2017–2022 et de 92,9 % (IC 95 % : 88,2-97) en 2010 à 0 % en 2021 pour le block 2. Les données parasitologiques et sérologiques confirment les résultats du suivi entomologique selon lesquels il est très probable que la population de *Glossina palpalis gambiensis* soit éradiquée des Niayes, et que la transmission de la TAA a été interrompue dans la zone traitée. Elles indiquent l’efficacité de l’approche adoptée, et montrent que la TAA peut être durablement éliminée grâce à la création d’une zone exempte de *G. p. gambiensis*.

## Introduction

Animal and human trypanosomoses are parasitic diseases caused by flagellate protozoa of the genus *Trypanosoma*, which are transmitted to mammalian hosts by blood-sucking tsetse flies (Diptera, Glossinidae), *i.e.*, the sole cyclical vectors of these parasites. African animal trypanosomosis (AAT) is one of the biggest constraints to the development of more sustainable and effective livestock production systems in the humid and sub-humid areas of West Africa [23]. More than 50 million cattle and nearly 100 million small ruminants are estimated to be at risk of becoming infected with the disease. In sub-Saharan Africa, the direct annual losses in livestock productivity combined with increased morbidity and mortality have been estimated at US $ 1.2 billion [23]. These economic figures increase to US $ 4.75 billion when indirect costs, such as loss in manure and reduced animal traction, are taken into account [8]. In addition, at least 35 million doses of trypanocidal drugs are administered to livestock each year at an annual cost of € 30 million [38]. Recently, a phased conditional pathway has been proposed for the control of AAT [15] and a phased conditional approach (PCA) for the management of tsetse flies [44]. These strategies, together with improved tools and methods to prioritize intervention areas, have facilitated the selection of the most appropriate intervention strategy (suppression versus eradication) taking into account environmental, epidemiological, and socioeconomic contexts [3, 7, 17]. Adequate management of AAT in Africa could entail annual potential benefits of more than US $ 700 million in terms of milk and meat productivity [26].

*Glossina palpalis gambiensis* Vanderplank was the only tsetse species present in the Niayes of Senegal. Its presence was closely linked with tree crops watered year-round and residual riparian vegetation [5, 17, 42]. Parasitological surveys in cattle carried out between 1965 and 1969 [43] showed a prevalence of 53% for *Trypanosoma vivax*, which prompted a first attempt in the 1970s to eradicate the *G. p. gambiensis* population from the Niayes. The project resulted in 10 years of supposed tsetse absence in the area, but in the 1990s, AAT re-surfaced in the Niayes and epizootiological surveys in cattle indicated a mean *T. vivax* prevalence of 9.9%. Initial entomological surveys revealed the presence of *G. p. gambiensis* from the Parc de Hann in Dakar in the west to Thiès, located more to the east. In addition, the observed infection risk was three times higher in the area infested by tsetse flies than in the area where tsetse flies were absent [31].

The Government of Senegal embarked in 2005 on a project entitled “Projet de lutte contre les glossines dans les Niayes” (Tsetse control project in the Niayes) with the goal to create a zone free of tsetse flies. Staff of the Direction de l’Élevage (DIREL) (now called Direction des Services Vétérinaires (DSV)) of the Ministry of Livestock and Animal Production and the Institut Sénégalais de Recherches Agricoles (ISRA) of the Ministry of Agriculture and Rural Equipment implemented the project. The project received technical and financial support from the International Atomic Energy Agency (IAEA), the Food and Agriculture Organization of the United Nations (FAO), the Centre de Coopération Internationale en Recherche Agronomique pour le Développement (CIRAD), and the US Department of State through the Peaceful Uses Initiative (PUI). The Centre International de Recherche-Développement sur l’Élevage en zone Sub-humide (CIRDES), Burkina Faso, the Slovak Academy of Sciences (SAS), Slovakia, and l’Institut de Recherche pour le Développement (IRD), France, were other full- or part-time partners in the project [44].

The project was implemented following area-wide integrated pest management (AW-IPM) principles [21, 25]. Control tactics included the deployment of traps and targets impregnated with deltamethrin [28], applications of insecticide pour-on formulation on livestock [20], sporadic ground spraying with insecticides in “hot spot” areas [24] and the release of sterile males [10, 46]. In addition, project implementation followed a PCA whereby implementation of the next phase is conditional to completion of all or most of the activities in the previous phase. For practical reasons, the project area was divided into three blocks that were treated sequentially [44].

A similar strategy was used to sustainably remove an isolated population of *Glossina austeni* Newstead from Unguja Island of Zanzibar [46]. The *G. p. gambiensis* population from the Niayes could be considered an “isolated island population” as it proved to be genetically isolated from the nearest tsetse population in the Sine Saloum located 200 km southwards from the Niayes [39]. In Senegal, the potential increase in animal sales following tsetse elimination in the Niayes area has been estimated at € 2800/km^2^/year [2].

As part of a series of baseline data collection activities (entomological surveys [5], tsetse population genetics [39], and socioeconomic [2] and environmental impact studies [9, 44]), parasitological and serological surveys were carried out to assess the disease prevalence in livestock [37]. A survey carried out in August to October 2007 in the Niayes and the Petite Côte revealed a serological prevalence of 28.7%, 4.4%, and 0.3% for *Trypanosoma vivax*, *T. congolense* and *T. brucei brucei*, respectively [37].

This paper presents the results of serological and parasitological prevalence surveys in cattle in the Niayes during the implementation of the AW-IPM eradication programme. The goal was to investigate whether the sustained removal of the sole cyclical vector would result in interruption of AAT transmission in cattle.

## Material and methods

### Ethics statement

The study was conducted in the framework of the tsetse eradication campaign in Senegal, implemented by the Direction des Services Vétérinaires, Ministry of Livestock and the ISRA (Institut Sénégalais de Recherches Agricoles)/LNERV, Ministry of Agriculture and Rural Equipment. This project received official approval from the Ministry of Environment of Senegal, under permit No. 0874/MEPN/DE/DEIE/mbf. Animals were sampled with the verbal consent of the cattle owners.

### Study site and activities implemented

**Figure 1:**
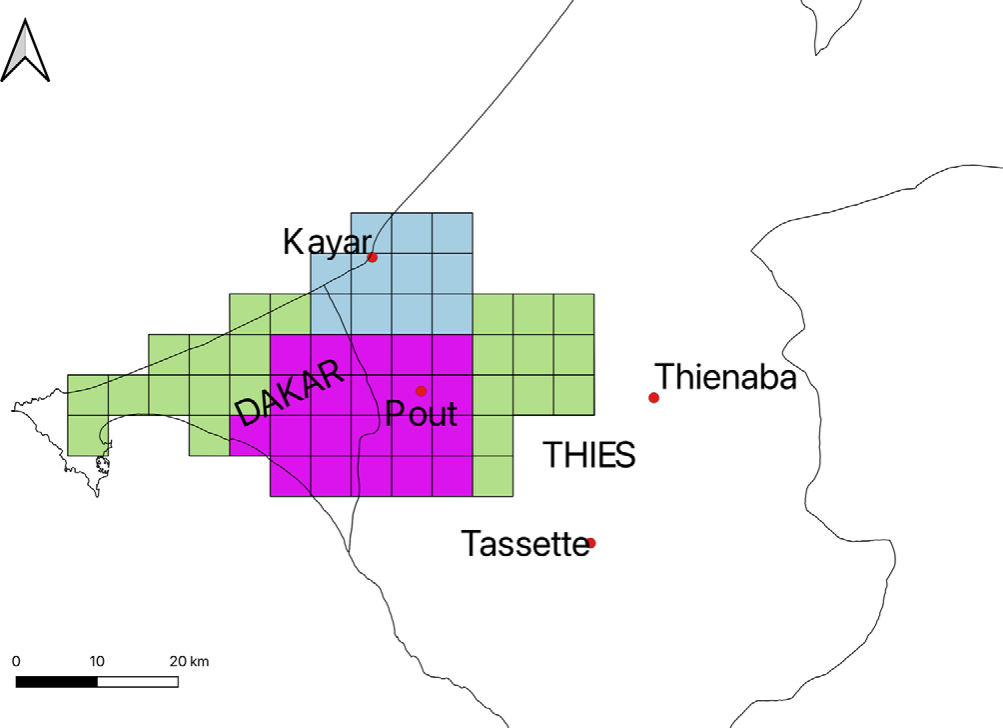
Map of Senegal with the location of the study area: study sites (red dots) and surface covered by block 1 (grid cells in light blue), block 2 (grid cells in purple) and block 3 (grid cells in green). Each grid cell represents an area of 25 square kilometres (5 × 5 km). The map was created using QGIS software v. 2.18.7 (http://www.qgis.org/fr/site/).

The Niayes area of Senegal has a special microclimate that is buffered by the influence of the Atlantic Ocean, and hence, is wetter and colder than the surrounding desert areas [43]. The lack of extreme hot and dry weather in the Niayes allows the development of intensive and semi-intensive farming systems with more productive exotic cattle breeds (particularly Holstein and Jersey) and cross-breeds [1]. Unfortunately, this unique ecosystem is also suitable to host *G. p. gambiensis*, the local vector of AAT. Using data from the entomological and veterinary baseline data surveys [5, 37], three sites were selected for this study: one outside the tsetse-infested area (block 0), a moderately infested area (block 1) and a highly infested area (block 2). Block 3, a low infestation area including Thiès, Parc de Hann and Sangalkam, was for economic reasons not included in this survey (Figure 1).

The village of Tassette was initially selected as an area outside of the tsetse belt but, due to the reluctance of farmers to collaborate with the sampling of blood from their livestock, this site was replaced in 2013 by Thiénaba, which was also located outside of the originally tsetse-infested zone.

**Figure 2:**
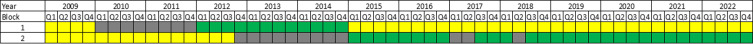
Implementation schedule of the area-wide integrated pest management (AW-IPM) programme in blocks 1 and 2 (2009-2022): in grey: suppression phase, in green: eradication phase (sterile fly releases) and in yellow: monitoring phase. Entomological monitoring was carried out throughout programme implementation.

Blocks 1, 2 and 3 were sequentially targeted by an AW-IPM programme (Figure 2) that included a sterile insect technique (SIT) component to eradicate the isolated tsetse populations. The IPM strategy that was selected comprised the suppression of the tsetse population with insecticide-impregnated traps/targets and the use of “pour-on” for cattle, followed by the release of sterile males to eliminate the remaining pockets.

Block 1 includes Kayar, a small coastal town located 58 km north of Dakar. The vegetation of the area consists of palm groves, citrus and mango orchards, and euphorbia hedges with swampy areas of permanent water that are used as drinking areas for livestock. The area was moderately infested with tsetse flies, with an apparent density of <1 fly/trap/day before the start of the AW-IPM programme. From 2010 to 2011, the fly population was suppressed using insecticide-impregnated traps and insecticide pour-on treatment of livestock, and this was followed in 2012 by releases of sterile male flies by air and from the ground. A total of 707,040 sterile males were released between 2012 and 2014. The last wild tsetse fly was trapped in August 2012, and the Kayar tsetse population is considered eradicated (Figure 2) [17].

Block 2 includes Pout, a municipality located in the region of Thiès and is located 54 km east of Dakar. This town is known for its production and trade of fruits and vegetables. The area was considered highly infested with tsetse flies which was exemplified by an apparent fly density of >10 flies/trap/day before the start of the suppression phase. Suppression activities started in December 2012 and lasted until 2014, and the release of sterile males was initiated in 2015. A total of 8,836,666 sterile males were released in the area from 2014 to 2022 (Figure 2). At the time of writing (2023), no wild flies have been trapped in the area since March 2021, with the exception of two virgin females caught in January 2022 which could not be considered a self-sustaining population as no further catches were obtained despite increased trapping intensity [40].

Entomological monitoring was carried out during the entire AW-IPM programme using 1–43 biconical or Vavoua traps in each grid cell of 25 km^2^ depending on the area of habitat suitable for tsetse flies, as described in [5]. In all 21 and 168 traps were installed in block 1 and block 2, respectively and followed up twice a month. These traps were used to catch the biological vector as well as mechanical vectors (Stomoxes and Tabanids).

### Blood collection and analyses

Sampling events were conducted once per year during the study period. At each event, an average of 100 cattle were sampled at each of the three sites, which corresponded to approximately 14–17% of the resident cattle population, considering a mean grazing radius of 5 km and a cattle density of 8–9/km^2^ in the survey area [2]. Each year, the sampling was conducted simultaneously at the three sites, during the dry season between March and July. In 2020, following the unexpected detection of *Trypanosoma spp.* in some animals in blocks 1 and 2 during the regular survey, the positive animals were screened again in November.

Blood was sampled in 5 mL gel & clot activator tubes, and sera were obtained by centrifugation at 1,500 rpm for 10 min and transferred into Nunc tubes for storage at −20 °C until their analysis. The Ab-ELISA technique [13] was used to determine the serological prevalence of different *Trypanosoma* species in the study area.

To determine the serological prevalence, antigens of *Trypanosoma brucei*, *T. congolense* and *T. vivax*, as well as specific positive sera directed against these antigens, were used. For each serum to be tested, the Ab-ELISA tests for *T. brucei*, *T. congolense* and *T. vivax* were carried out simultaneously. The most likely trypanosome species was inferred based on the result of the Ab-ELISA test with the highest relative percentage of positivity among the three tests [13]. A serum was considered positive for *Trypanosoma spp*. if it was positive in at least one of the three Ab-ELISA tests. In the present study, the results are expressed in terms of pan-trypanosome seroprevalence and not in terms of the seroprevalence of the particular *Trypanosoma spp.* used as antigen donor in the Ab-ELISA tests due to the limited specificity of this method. *Trypanosoma spp*. antigens and positive controls were obtained from CIRDES/CIRAD-Intertryp that are designated reference laboratories for AAT by the World Organisation for Animal Health (WOAH).

For the parasitological analyses, the blood was collected in capillary tubes and centrifuged at 1,500 rpm for 5 min. After centrifugation, the capillary tube was cut 1 mm below the buffy coat (white clot/erythrocyte interphase) using a diamond-tipped pencil to include the upper layer of red blood cells. After homogenisation by mixing, the contents were mounted between slide and coverslip and observed under a microscope. The buffy coat technique is a direct microscopic method used to detect trypanosomes, identify their species and assess the level of parasitaemia [4].

Since 2020, to confirm the presence/absence of the parasite, we developed a PCR test to assess presence of the trypanosome genome in blood samples. Genomic DNA was extracted from 200 μL of Buffy coat using a QIAamp DNA Mini Kit (QIAGEN, Hilden, Germany), according to the manufacturer’s instructions and maintained at −20 °C until further use. The ITS1 rDNA primers (forward 5′-CCGGAAGTTCACCGATATTG-3′ and reverse 5′-TTGCTGCGTTCTTCAACGAA-3′) [33] were used to identify all *Trypanosoma* species. The pairs of primers TBR1: 5′-CGAATGAATATTAAACAATGCG-CAG-3′/TBR2: 5′-AGAACCATTTATTAGCTTTGTTGC-3′ [29] and EVA1: 5′-ACATATCAACAACGACAAAG-3′/EVA2: 5′-CCCTAGTATCTCCAATGAAT-3′ [32] were used to confirm species from *Trypanozoon* subgenus. The PCR was carried out in 25-μL reaction mixtures containing Taq 5X Master Mix, 10 μM of each primer and 3 μL of extracted DNA. The PCR cycling conditions were as follows: an initial denaturation step at 95 °C for 1 min, followed by 35 cycles of 95 °C for 30 s, 58 °C for ITS1 rDNA primers or 55 °C for TBR1-2 and EVA1-2 primers for 30 s, 72 °C for 60 s, and final extension at 72 °C for 10 min. The PCR products were visualised on 1.5% agarose gels with a Gel Red staining after migration of 90 min at 100 volt by electrophoresis. The species were identified according to the PCR product band sizes indicated in Njiru *et al.* [33]: 480 bp for members of subgenus *Trypanozoon* (*T. brucei brucei*, *T. evansi*, *T. b. rhodesiense* and *T. b. gambiense*); 700 bp for *T. congolense*, savannah; 250 bp for *T. vivax*, or 177 bp for *T. brucei brucei* as indicated in Moser *et al*. [29] or 138 bp for *T. evansi*. In addition, farmers were asked questions related to herd movements to determine the probable origin of unexpected infections.

### Data analyses

The parasitological prevalence was calculated as the proportion of positive blood samples for *Trypanosoma spp.* (all species together) out of the total number of blood samples collected at one site during one sampling event (year). The pan-trypanosome serological prevalence was calculated as the proportion of *Trypanosoma spp.* positive sera out of the total number of sera collected at one site during one sampling event. From this value, the true prevalence was calculated taking into account the sensitivity and specificity values of the Ab-Elisa test according to Desquesnes et al [13], Sp > 96% and Se > 92%. The binomial confidence intervals for the true prevalence were calculated using the Normal approximation method.

Logistic regression was used to analyse the data. A negative binomial regression model (NBRM) [22, 27] was used to assess seroprevalence as a function of time and space. Time is the year rank starting at the beginning of the period: 1 for 2009, 2 for 2010, 3 for 2011, *etc*. Time is considered the integer. Space is represented by the Block where data were collected. Factor levels are 0, 1 and 2 for Block 0, Block 1 and Block 2, respectively. NBRM has been widely adopted for regression of count responses because of its convenience to take into account over dispersion. *y* represents the univariate count response variable and *X* the *p*-dimensional vector of known explanatory variables. The model is:

$$y|X\;∼\;\mathrm{Poisson}\left(\lambda \right),$$

where *λ* follows a Gamma distribution of mean μ and the mean and variance are given by

$$E\left[y\right]=\;\mu \;\mathrm{and}\;\mathrm{Var}\left[y\right]=\mu +\frac{\mu^{2}}{k},$$

where *k*, is an additional parameter called the dispersion parameter calculated by the model.

The model goodness of fit was evaluated using the Pearson chi-squared *χ*^2^ statistics calculated by:

$$\chi^{2}\;=\sum_{i=1}^{N}\frac{{\left(y_{i}-\;\mu \right)}^{2}}{\mathrm{Var}\left[y_{i}\right]}.$$

Assuming that *N* is the total number of observations.

The mean apparent density of *G. p. gambiensis* and mechanical vectors was computed as the mean number of flies in a given trap on a given day during the sampling period.

All of the analyses were carried out with R software [35]; the aods3 package was used to fit models and model curves were done using ggplot2 package.

## Results

From 2009 to 2022, a total of 4,359 blood samples were collected on regular sampling sessions from livestock at the three sites (Table 1) and analysed with the buffy coat technique and the Ab-ELISA. With respect to direct parasitological examination, no animal was found to be positive after 2019 for three consecutive years (2020–2022) in both the non-infested area and the previously tsetse-infected areas. Positive cases detected before implementation of the AW-IPM programme were already very scarce (Table 1).

**Table 1:** Yearly serological and parasitological prevalences of animal trypanosomosis in the Niayes area from 2009 to 2022 and number of PCR-positive samples from 2020 to 2022; *CI = confidence interval.

Year	Site	Number of samples	Number of seropositive samples	Apparent seroprevalence (%)	True seroprevalence (%)	95% CI of the true seroprevalence (%)	Number of samples positive in buffy coat test	Parasitological prevalence (%)	Number of PCR-positive samples
2009	Pout	126	75	59.5	63.1	54.6–71.6	N/A	N/A	N/A
	Kayar	102	21	20.6	18.9	11.2–26.5	N/A	N/A	N/A
	Tassette	132	7	5.3	1.5	0.0–3.6	N/A	N/A	N/A
2010	Pout	119	102	85.7	92.9	88.2–97.5	N/A	N/A	N/A
	Kayar	101	16	15.8	13.5	6.8–20.2	N/A	N/A	N/A
	Tassette	133	1	0.8	0	0.0–0.0	N/A	N/A	N/A
2011	Pout	114	50	43.9	45.3	36.1–54.5	0	0	N/A
	Kayar	97	3	3.1	0	0.0–0.1	0	0	N/A
	Tassette	94	3	3.2	0	0.0–0.1	5	4.4	N/A
2012	Pout	105	19	18.1	16	9.0–23.1	0	0	N/A
	Kayar	76	3	3.9	0	0.0–0.1	3	3.9	N/A
2013	Pout	111	12	10.8	7.7	2.7–12.8	2	1.8	N/A
	Kayar	89	2	2.3	0	0.0–0.1	3	3.4	N/A
	Thiénaba	100	1	1	0	0.0–0.1	0	0	N/A
2014	Pout	112	9	8	4.6	0.7–8.5	2	1.8	N/A
	Kayar	102	1	0.98	0	0.0–0.0	4	3.0	N/A
	Thiénaba	100	1	1	0	0.0–0.1	1	1	N/A
2015	Pout	103	17	16.5	14.2	7.4–21.0	0	0	N/A
	Kayar	86	11	12.8	10	3.6–16.4	0	0	N/A
	Thiénaba	96	3	3.1	0	0.0–0.1	0	0	N/A
2016	Pout	116	8	6.9	3.3	0.0–6.6	0	0	N/A
	Kayar	100	1	1	0	0.0–0.1	0	0	N/A
	Thiénaba	199	9	4.5	0.6	0.0–1.7	0	0	N/A
2017	Pout	129	9	7	3.4	0.2–6.5	0	0	N/A
	Kayar	94	0	0	0	0.0–0.1	0	0	N/A
	Thiénaba	100	1	1	0	0.0–0.1	0	0	N/A
2018	Pout	103	9	8.7	5.4	1.0–9.8	0	0	N/A
	Kayar	103	0	0	0	0.0–0.0	2	1.9	N/A
	Thiénaba	100	2	2	0	0.0–0.1	0	0	N/A
2019	Pout	99	10	10.1	6.9	1.9–12.0	1	1	N/A
	Kayar	74	0	0	0	0.0–0.1	0	0	N/A
	Thiénaba	118	7	5.9	2.2	0.0–4.9	0	0	N/A
2020	Pout	99	15	15.2	12.7	6.1–19.3	0	0	1
	Kayar	102	10	9.8	6.6	1.7–11.5	0	0	0
	Thiénaba	100	8	8	4.5	0.4–8.7	0	0	0
2021	Pout	99	1	1	0	0.0–0.1	0	0	0
	Kayar	105	0	0	0	0.0–0.0	0	0	0
	Thiénaba	102	1	1	0	0.0–0.0	0	0	0
2022	Pout	121	3	2.5	0	0.0–0.0	0	0	0
	Kayar	96	0	0	0	0.0–0.1	0	0	0
	Thiénaba	100	1	1	0	0.0–0.1	0	0	0

During the pre-implementation period of the AW-IPM programme (2009 and 2010), a pan-trypanosome seroprevalence of 18.9% (with a 95% confidence interval (CI): 11.2–26.5) and 92.9% (95%CI: 88.2–97) was recorded in Kayar and Pout, respectively. During the implementation of the AW-IPM programme from 2011 to 2022, a decrease in the seroprevalence was observed in Kayar and Pout from 10% (95%CI: 3.6–16.4) in 2015 to 0% (95%CI: 0–0.1) in 2022 in Kayar and from 45.3% (95%CI: 36.1–54.5) in 2011 to 0% in 2021 in Pout. Small variations in the seroprevalence were observed in the non-infested sites (Tassette and Thiénaba) during the entire survey period (2009–2022), but the seroprevalence never exceeded 4.5%. Similarly, moderate variations of the seroprevalence were also noted in Pout and Kayar. Secondary prevalence peaks were observed in Pout (seroprevalence of 14.2%, 95%CI [7.4–21.0%] in 2015 and seroprevalence of 12.7%; 95% CI [6.1–19.3%] in 2020) as well as in Kayar in 2020 (seroprevalence of 6.6%; 95% CI [1.7–11.5]). At both sites, the seroprevalence dropped again to zero from 2020 to 2022 (Table 1). In 2020, positive samples collected from the regular sampling session were confirmed in the November session in Pout (12 positives out of 14; 1 died) and Thiénaba (8 positives out of 8), but not in Kayar where all 10 samples were negative. Regarding PCR analysis, samples from Kayar and Thiénaba were negative for all *Trypanosoma* species tested. Only one sample was positive for *Trypanosoma brucei brucei* in Pout in 2020.

**Figure 3:**
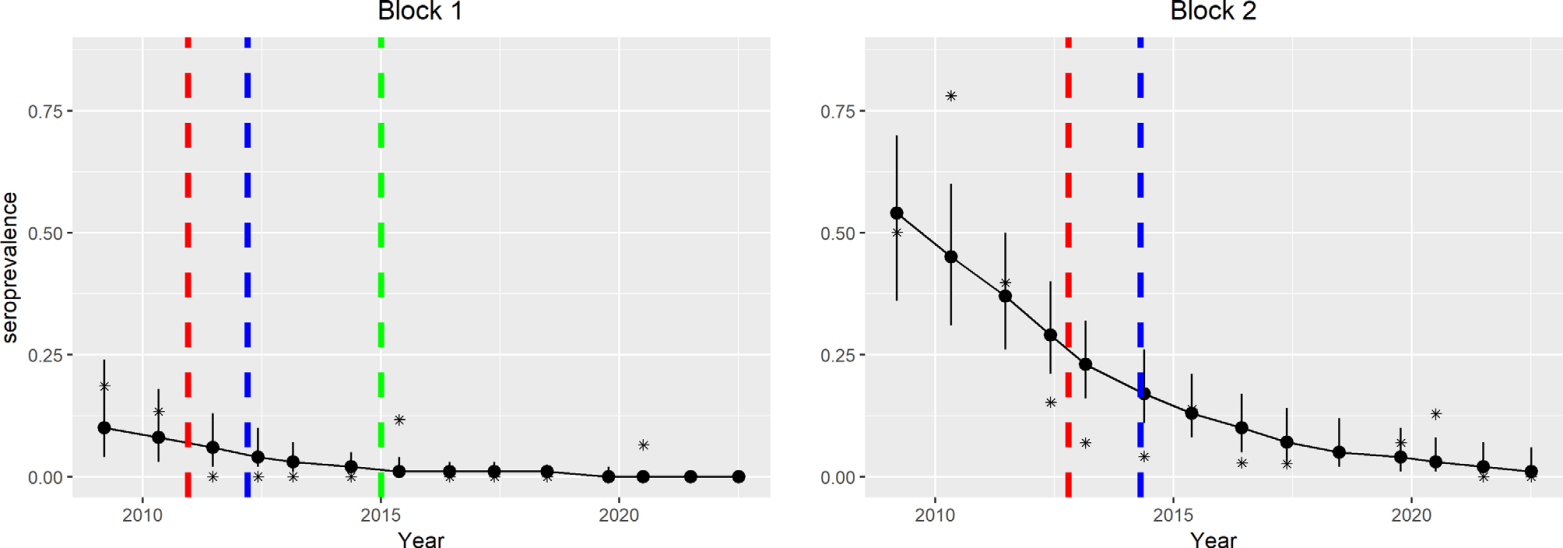
Annual seroprevalence predicted by the negative binomial regression model in blocks 1 and 2 during the implementation of the AW-IPM programme, with prediction intervals (red dotted line: start of the suppression phase; blue dotted line: start of the eradication phase; green dotted line: start of the monitoring phase).

The negative binomial regression model described well the temporal decrease in seroprevalence (Figure 3) with a high goodness-of-fit (*χ*^2^ = 54.1684, *df* = 34, *p* = 0.01541) (Table 2). The regression model assumed a linear temporal trend, which smoothed out the secondary peaks observed in the annual data.

**Table 2:** Negative binomial regression model (NBRM) used to assess seroprevalence as a function of time and space (*** = significant).

	Regression coefficient	Standard error	*z*-value	*p*-value
Block 0	−3.1101	0.5687	−5.4684	4.541e−08***
Block 1	−1.5425	0.4187	−3.6839	0.0002***
Block 2	0.3454	0.2825	1.2228	0.2214
Time	0.0029	0.0699	0.0410	0.9673
Block 1: time	−0.3193	0.1194	−2.6746	0.0075***
Block 2: time	−0.3183	0.0901	−3.5350	0.0004***

**Figure 4:**
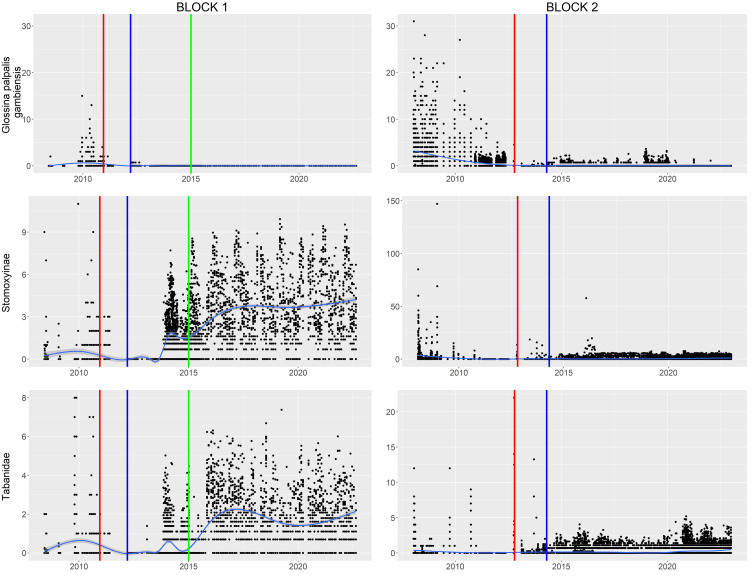
Apparent densities of vectors (*Glossina palpalis gambiensis*, Stomoxyinae and Tabanidae) in blocks 1 and 2 (Red vertical line: start of the suppression phase, blue vertical line: start of the eradication phase, green vertical line: stop of the eradication phase). The light blue curve represents a smoothing estimation.

Entomological results derived from monthly or biweekly monitoring in Kayar indicated that the last indigenous tsetse fly was trapped in August 2012; therefore, Kayar was considered a tsetse-free zone. Since the implementation of the AW-IPM programme, the trap catches of tsetse flies in Pout have been very low (0.329 ± 1.192) and are nil during the last year (Figure 4). On the other hand, the apparent density per trap of mechanical vectors, notably Stomoxyinae has increased significantly in Kayar (*p* = 0.0006) with a percentage increase of 62,361.21% between the beginning and end of the study period. Simultaneously the average density of Tabanids has increased along the study in Kayar but in lower proportions (*p* = 0.0293) with a percentage increase of 295.08% (Table 3).

**Table 3:** Apparent density (number of flies per trap per day) of mechanical vectors in the infested areas from 2013–2022.

	Year	2013	2014	2015	2016	2017	2018	2019	2020	2021	2022
Kayar											
Stomoxes	Mean	0.0629	3.2746	8.0582	9.7847	16.7734	13.5857	18.4897	16.7480	20.3504	39.2881
	SD	0.5311	12.5887	35.2851	29.4209	42.4801	44.2963	57.1485	32.7945	55.9415	192.7126
Tabanids	Mean	0.1239	0.1581	0.2428	1.0996	0.7082	0.7082	1.0550	0.7877	0.5072	0.4895
	SD	0.8723	0.7369	1.1675	2.3162	1.4844	2.9882	3.9876	1.5093	1.2162	1.2124
Pout											
Stomoxes	Mean	NA	0.0481	0.0635	0.1409	0.2080	0.1613	0.3750	0.2290	0.4472	0.4600
	SD	NA	0.4173	0.8576	1.1444	1.5602	1.5437	3.5722	1.9396	1.4521	1.8929
Tabanids	Mean	NA	0.0238	0.0204	0.0309	0.0121	0.0046	0.0070	0.0532	0.0536	0.0344
	SD	NA	0.0878	0.1312	0.1258	0.0555	0.0317	0.0411	0.3740	0.2110	0.1053

## Discussion

The application of the AW-IPM strategy in Kayar of the Niayes resulted in local eradication of the *G. p. gambiensis* population in 2012. Consequently, a significant decrease in the seroprevalence of trypanosomosis in Kayar was observed and, with the exception of 2015, this prevalence has been similar to that observed in the non-tsetse infested areas corresponding to absence of transmission. Surveys conducted in 2020 following the unexpected detection of positive animals in Kayar revealed that some herds had moved to areas that were still infested with tsetse flies in block 2 to take advantage of available grazing areas when the local pastures were depleted. This practice is common among livestock owners during the dry season to reduce livestock mortality and may explain the positive cases observed in Kayar where the tsetse population had been eradicated since 2012. The dynamics of AAT depends both on the eco-epidemiological cycle and also on certain practices such as grazing on natural pastures or transhumance [1]. The occurrence of some residual prevalence in areas where tsetse flies have been eliminated and in the area outside the tsetse belt is then related to the importation of trypanosomes through animal movements from tsetse-infested areas, and local transmission by mechanical vectors [30, 34].

Alternatively, residual cases may be false positives related to the specificity of the ELISA test. A similar trend was observed in Pout until 2020, *i.e.*, a significant decrease in the temporal seroprevalence of trypanosomosis; however, the fact that the tsetse populations were not yet eradicated over the entire block and only suppressed to very low levels translated in an albeit low but still higher prevalence than in the non-tsetse infested areas. This indicates and confirms that a very low density of a *G. p. gambiensis* population can maintain trypanosome transmission.

In Pout, the small increase in the prevalence in 2015 and 2020 was probably due to cyclical transmission by the tsetse flies that were still present at low densities in the area. However, in Kayar, a similar peak in the prevalence was observed in 2015 in the absence of any tsetse population (eradicated since 2012). One possibility might be the involvement of biting flies such as Tabanidae and Stomoxyinae, that can transmit *Trypanosoma spp.* in the absence of tsetse flies as reported in several areas of West Africa [11, 12, 14]. This interpretation is supported by the species-specific analysis results which showed that the 2015 peak in Kayar was caused by *T. vivax*, while the 2020 peak was due to *T. brucei* (data not shown). We have no firm explanation with respect to the increase in densities of Stomoxyinae and Tabanidae, but is unlike to be due to competitive release, which occurs when a species benefits from the reduction of its competitor. The larval habitats of these biting flies are totally different from those of tsetse flies, whose larvae do not feed at all in the environment, and whereas mechanical vectors can reduce the blood feeding success of adult tsetse, the reverse is not likely [41]. The observed trend might be due to climatic factors or more likely to the intensification of cattle farming systems, as a consequence of the removal of AAT [2]. Dairy farms provide perfect larval environments for Stomoxyinae, hence their common name of “stable flies” and lead to their proliferation in Reunion island [6, 18]. Similarly, horse farms have also increased in the target area and might favour Tabanidae, also named “horse flies”. Transmission by mechanical vectors, however, requires certain conditions: (i) high parasitaemia in infected animals; (ii) a high density of potential mechanical vectors; (iii) high susceptibility of a large proportion of the potential population at risk; and (iv) close contact between susceptible animals and infected animals. From our results, it seems clear that these conditions do not currently prevail in the Niayes.

The overall low parasitaemia in the Niayes is related to the presence of Diakoré, a cattle breed mixed with the Ndama trypanotolerant breed, and may explain the low number of positive parasitological cases, whereas the seroprevalence was quite high, at least at the beginning of the AW-IPM programme. In addition, the buffy coat method is known to have a low sensitivity in comparison with serological techniques [19].

*Glossina palpalis gambiensis* was the only *Glossina* species present in the Niayes and hence, solely responsible for the cyclic transmission of the pathogenic trypanosomes. The parasitological and serological results obtained in 2021 and 2022 in all the blocks showed that transmission of trypanosomes by the biological vector has stopped. This confirms the data from the entomological monitoring system set up in the framework of the project. Pending the upcoming technical declaration of tsetse fly elimination from the Niayes, we can confirm that the implementation of the project following the area-wide integrated pest management (AW-IPM) principles has provided strong arguments to support the declaration of freedom from the tsetse fly and AAT in the Niayes.

The data presented in this paper are very similar to those obtained in the eradication programme of *Glossina austeni* on Unguja Island of Zanzibar. Like in the Niayes, the removal of the *G. austeni* population (the island was declared free of tsetse in 1997) resulted in an interruption of the cyclical transmission of AAT, and the disease disappeared. Despite the presence of high population densities of Stomoxyinae, mechanical transmission could not sustain the transmission of the disease [36, 45].

It will be necessary to continue monitoring trypanosome prevalence in the Niayes after eradication of the tsetse population, to confirm the sustained trypanosomosis-free status in all cattle in the Niayes [16].
